# Local anaesthetic infiltration for peri-operative pain control in total hip and knee replacement: systematic review and meta-analyses of short- and long-term effectiveness

**DOI:** 10.1186/1471-2474-15-220

**Published:** 2014-07-05

**Authors:** Elsa MR Marques, Hayley E Jones, Karen T Elvers, Mark Pyke, Ashley W Blom, Andrew D Beswick

**Affiliations:** 1 School of Social and Community Medicine, University of Bristol, Canynge Hall, 39 Whatley Road, Bristol BS8 2PS, UK; 2 Musculoskeletal Research Unit, School of Clinical Sciences, University of Bristol, Avon Orthopaedic Centre, Southmead Hospital, Bristol BS10 5NB, UK; 3 North Bristol Healthcare Trust, Southmead Hospital, Westbury-on-Trym, Bristol BS10 5NB, UK

**Keywords:** Hip replacement, Knee replacement, Anaesthesia, Systematic review, Meta-analysis

## Abstract

**Background:**

Surgical pain is managed with multi-modal anaesthesia in total hip replacement (THR) and total knee replacement (TKR). It is unclear whether including local anaesthetic infiltration before wound closure provides additional pain control.

**Methods:**

We performed a systematic review of randomised controlled trials of local anaesthetic infiltration in patients receiving THR or TKR. We searched MEDLINE, Embase and Cochrane CENTRAL to December 2012. Two reviewers screened abstracts, extracted data, and contacted authors for unpublished outcomes and data. Outcomes collected were post-operative pain at rest and during activity after 24 and 48 hours, opioid requirement, mobilisation, hospital stay and complications. When feasible, we estimated pooled treatment effects using random effects meta-analyses.

**Results:**

In 13 studies including 909 patients undergoing THR, patients receiving local anaesthetic infiltration experienced a greater reduction in pain at 24 hours at rest by standardised mean difference (SMD) -0.61 (95% CI -1.05, -0.16; p = 0.008) and by SMD -0.43 (95% CI -0.78 -0.09; p = 0.014) at 48 hours during activity.

In TKR, diverse multi-modal regimens were reported. In 23 studies including 1439 patients undergoing TKR, local anaesthetic infiltration reduced pain on average by SMD -0.40 (95% CI -0.58, -0.22; p < 0.001) at 24 hours at rest and by SMD -0.27 (95% CI -0.50, -0.05; p = 0.018) at 48 hours during activity, compared with patients receiving no infiltration or placebo. There was evidence of a larger reduction in studies delivering additional local anaesthetic after wound closure. There was no evidence of pain control additional to that provided by femoral nerve block.

Patients receiving local anaesthetic infiltration spent on average an estimated 0.83 (95% CI 1.54, 0.12; p = 0.022) and 0.87 (95% CI 1.62, 0.11; p = 0.025) fewer days in hospital after THR and TKR respectively, had reduced opioid consumption, earlier mobilisation, and lower incidence of vomiting.

Few studies reported long-term outcomes.

**Conclusions:**

Local anaesthetic infiltration is effective in reducing short-term pain and hospital stay in patients receiving THR and TKR. Studies should assess whether local anaesthetic infiltration can prevent long-term pain. Enhanced pain control with additional analgesia through a catheter should be weighed against a possible infection risk.

## Background

Total hip replacement (THR) and total knee replacement (TKR) are widely used to treat diseased and damaged joints. In 2012 there were 75,366 primary THR and 76,497 primary TKR procedures recorded in the National Joint Registry for England, Wales and Northern Ireland [1]. In the USA in 2010, the estimated numbers of THR and TKR procedures performed were 332,000 and 719,000, respectively [2].

Pain is the primary indication for THR and TKR and many preparatory, surgical and rehabilitation strategies target reduction in pain. However, both short- and long-term pain after THR and TKR are common [3-5]. Peri-operative pain is managed with multi-modal analgesia with additive or synergistic effects [6]. Regimens aim to achieve good pain relief immediately after surgery while allowing for early mobilisation and hospital discharge. Other methods such as spinal and epidural anaesthetics and the use of opioids may preclude early mobilisation and rehabilitation [7,8].

Pain management by infusion of local anaesthetic into wounds has been evaluated in diverse surgical procedures. In their systematic review, Liu and colleagues noted improved pain, reduced opioid use and side effects, increased patient satisfaction, and shorter hospital stay in patients receiving local anaesthetic infiltration [9]. Only one study included patients with THR or TKR [10], but further evaluations have been reported [11]. More recent meta-analyses in abdominal surgery [12], and lumbar spine surgery [13], have questioned the clinical value of local anaesthetic wound infiltration.

Using systematic review methods and meta-analysis, our objective was to synthesise evidence from randomised controlled trials (RCTs) evaluating the effectiveness of peri-operative local anaesthetic infiltration for pain control in patients with THR and TKR. Pain outcomes were considered along with post-operative opioid requirement, mobilisation, hospital stay and complications.

## Methods

We identified RCTs using methods described in the Cochrane handbook of systematic reviews of interventions [14]. The review was conducted in accordance with PRISMA guidelines [15] and a checklist is included as Additional file 1. This review builds on a previous literature review, without a further formal protocol published [11].

### Search strategy and selection criteria

We searched MEDLINE and Embase on OvidSP and Cochrane CENTRAL to 11^th^ December 2012. The search strategy covered RCTs, anaesthesia and analgesia, and THR and TKR terms (Table 1). We tracked citations of key articles in ISI Web of Science [10,16-19], and checked reference lists. Two reviewers scanned abstracts and titles, acquired potentially relevant articles, and decided on inclusion based on pre-specified criteria, with disputes resolved by other authors.

**Table 1:** Search strategy as applied in MEDLINE

1	Anesthetics, Local/or local anaesthetic.mp.
2	Anesthetics, Local/or Anesthesia, Local/or Local anaesthesia.mp.
3	Anesthetics/or Anesthesia/or anaesthesia.mp. or Anesthetics, Local/or Anesthesia, Local/
4	anesthesia.mp.
5	anaesthetic.mp.
6	amides.mp. or Amides/
7	(“Huneke neural therapy” or “Neural therapy of Huneke” or benzocaine or bensokain or “Aminobenzoic Acid” or “Aminobenzoate” or bupivacain* or buvacaina or sensorcaine or marcain* or svedocain* or levobupivacaine or carticain* or articain* or dibucaine or cinchocaine or Cincain or Nupercain* or Sovcaine or etidocaine or duranest or “W19053” or “W 19053” or “W-19053” or Lidocaine or Lignocaine or Octocaine or Xylesthesin or Xylocaine or Dalcaine or Xylocitin or Xyloneural or Mepivacain* or Carbocaine or Polocaine or isocaine or isogaine or Scandicain* or prilocaine or Propitocaine or Tetracaine or Tetrakain or Amethocaine or Dicaine or Pantocaine or Pontocaine or Trimecaine or Mesocaine or ropivacaine).mp. [mp = title, abstract, original title, name of substance word, subject heading word, protocol supplementary concept, rare disease supplementary concept, unique identifier]
8	1 or 2 or 3 or 4 or 5 or 6 or 7
9	(incision or port* or (surg* and wound)).mp. [mp = title, abstract, original title, name of substance word, subject heading word, protocol supplementary concept, rare disease supplementary concept, unique identifier]
10	acetabular.mp.
11	infiltration.mp.
12	wound infiltration.mp.
13	wound catheter.mp.
14	peri-articular.mp.
15	periarticular.mp.
16	intraarticular.mp.
17	intra-articular.mp.
18	9 or 10 or 11 or 12 or 13 or 14 or 15 or 16 or 17
19	Arthroplasty, Replacement, Knee/or Arthroplasty, Replacement, Hip/
20	exp Arthroplasty, Replacement, Hip/or exp Hip Prosthesis/ or hip replacement.mp.
21	exp Arthroplasty, Replacement, Knee/or exp Knee Prosthesis/or knee replacement.mp.
22	knee prosthesis.mp. or exp Knee Prosthesis/
23	hip prosthesis.mp. or exp Hip Prosthesis/
24	total hip.tw.
25	total knee.tw.
26	Orthopedic Procedures/ or orthopaedic surgery.mp.
27	19 or 20 or 21 or 22 or 23 or 24 or 25 or 26
28	8 and 18 and 27

*Ovid truncation character.

We included RCTs of patients with primary unilateral THR or TKR receiving local anaesthetic infiltration before wound closure compared with patients receiving no local anaesthetic infiltration or placebo. We also included studies comparing local anaesthetic infiltration with other forms of analgesia and studies with additional post-wound closure delivery of analgesics through catheters and injections. We excluded studies with interventions exclusively after wound closure and studies in patients receiving hip hemiarthroplasty or unicompartmental TKR. No language restrictions were applied and translations were made by colleagues as required.

### Data collection and extraction

Articles and inclusion/exclusion decisions were catalogued in Endnote X5. Data were extracted on to piloted forms and an Excel spreadsheet in duplicate. Authors were contacted for unpublished outcomes and missing data.

Information was extracted on study characteristics; participant characteristics; anaesthesia procedures common to randomised groups; intervention (content of infiltrate, timing and volume); additional intervention group treatments; and control group treatment including placebo and alternative analgesia regimens.

### Outcomes

Outcomes studied were pain at rest or during activity at 24 and 48 hours after surgery, opioid consumption, mobilisation, length of hospital stay in days, and long-term pain and function. Serious complications recorded were altered state of consciousness, cardiovascular complications requiring treatment, central nervous system toxicity, dysarthria, dyspnoea, major surgical complications, pneumonia, pulmonary embolism, respiratory depression, seizures, and swollen knee; or complications relating to deep infection. Adverse events were vomiting and nausea.

### Study quality

Potential sources of bias were recorded in a Cochrane risk of bias table [14]. We considered random sequence generation, allocation concealment, blinding of participants and personnel, blind outcome assessment, incomplete outcome data, selective reporting, and other sources of bias. We classified overall quality as low, unclear or high risk of bias.

### Meta-analysis

We conducted meta-analyses for pain at rest and during activity at 24 and 48 hours, length of hospital stay, and complications. Data collected on mobilisation, long-term pain and function outcomes were not suitable for meta-analyses and results were summarised using a descriptive narrative.

Follow-up times were approximated to the closest timing. When not specified, we assumed measurements were taken at rest. Analyses were carried out in Stata 12 and Review Manager 5 and results are reported with 95% confidence intervals. Funnel plots were inspected to assess small study effects [20]. Given the number of potential effect modifiers, we used random effects models for all meta-analyses.

In meta-analysis, means and standard deviations of continuous variables such as pain intensity are required for intervention and control groups. Pain outcomes are sometimes reported as medians and inter-quartile ranges due to the recognised ceiling effects of pain measures after successful pain management. However this is less of an issue during early recovery. Kerr and Kohan presented distributions of pain intensity scores at rest and during walking on the first and second day after THR or TKR [19]. The proportion of people reporting no pain, and thus reflecting a ceiling effect, ranged from 2 to 35% on days one and two and pain intensities showed near normal distributions.

Assuming a normal distribution, we estimated means and standard deviations from medians and inter-quartile ranges [14]. If no measures of variability were available in articles, we contacted authors to obtain standard deviations. If unavailable we used the method described by Walter and Yao to estimate standard deviations from ranges [21], or imputed values from the average across studies with the same outcome.

As pain scores are reported on different scales we used the standardised mean difference (SMD) as our measure of treatment effect in meta-analyses [22]. To help in the interpretation of the pooled estimates, we multiplied SMD values by the mean standard deviation on the widely reported 100 point VAS scale for the outcome. As the use of this method is entirely dependent on the chosen “typical” value [23], we used a mean standard deviation calculated from all studies reporting the outcome [14].

For length of hospital stay, we compared means and medians in studies reporting both, and examined individual level data provided by some authors. Distributions were right-skewed and followed a lognormal distribution. Some studies reported means and standard deviations directly. For studies that reported medians and inter-quartile ranges, or ranges, we estimated means and standard deviations on the log scale and then back-transformed them to the natural (unlogged) scale [24]. We reported the mean difference (MD) in days as our measure of treatment effect in meta-analyses. Complications were compared between randomised groups using meta-analysis with summary statistics calculated as the Peto odds ratio (OR), the method of choice when event rates are low [14,25].

### Analgesia regimen comparisons

**Figure 1:**
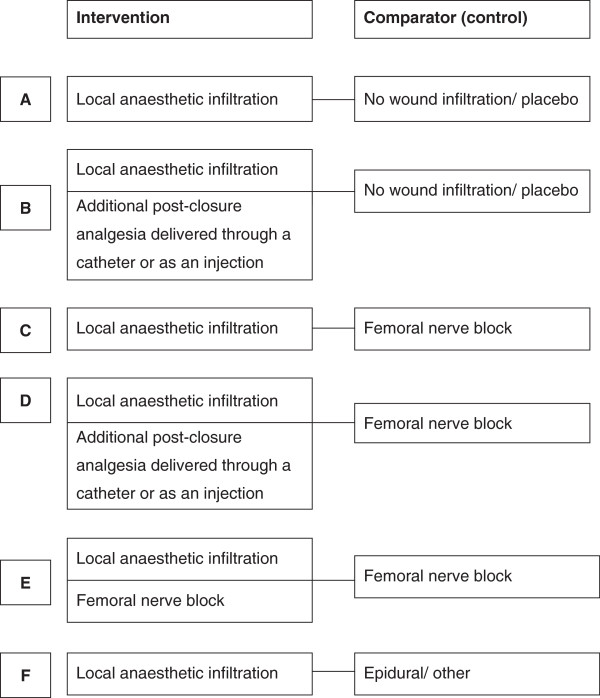
Wound infiltration anaesthesia regimens for interventions and comparators (controls).

Not all studies compared a local anaesthetic infiltration intervention with no intervention or placebo. Thus meta-analyses are reported separately for different regimen comparisons, which we label A-E. These are summarised in Figure 1.

THR studies were grouped into comparisons A and B; we and also report the combined comparison (A + B).

For TKR studies, we initially report results from a combined meta-analysis across the first two subgroups (A + B), comparing local anaesthetic infiltration with or without further post-closure intervention against control. Further analyses report the comparisons C, D and E.

### Heterogeneity and subgroup analyses

We quantified the differences in treatment effects between groups using meta-regression. Heterogeneity within meta-analyses was quantified using the τ^2^ and I^2^ statistics [26]. Sensitivity and sub-group analyses explored risk of bias in the study, use of additional analgesia delivered through a catheter or injection, and inclusion of non-steroidal inflammatory agents or steroids in the infiltrate.

## Results

**Figure 2:**
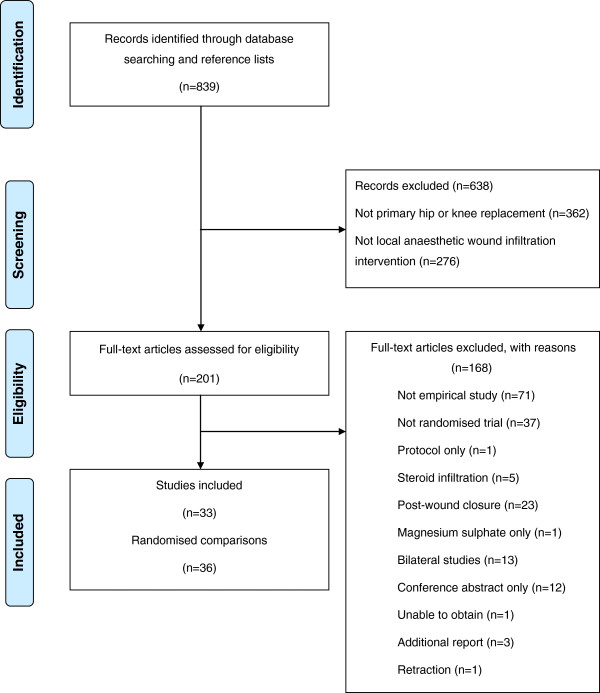
Systematic review flow diagram.

Searches identified 839 articles of which 33 described 36 RCTs evaluating local anaesthetic infiltration in THR or TKR. The flow diagram in Figure 2 summarises review progress. Some consistency in outcome reporting was apparent for pain outcomes but for opioid consumption and ambulation the variety of outcomes precluded meta-analysis.

### Small study effects

Inspection of funnel plots for each meta-analysis gave no strong indication of publication bias or small study effects, but numbers of studies in individual analysis groups were small such that it was difficult to assess asymmetry.

### Total hip replacement

Details of 13 studies including 909 patients with THR [16,27-37], or a large majority with THR [10], are summarised in Table 2 which also includes our summary risk of bias assessment. A more detailed assessment of risk of bias is included in Additional file 2. The mean number of patients randomised was 70 (range 37–120). We assessed that 10 studies were at low risk of bias while three studies had unclear risk of bias due to uncertainty about blinding of outcome assessments.

**Table 2:** Randomised controlled trials of local anaesthetic infiltration in total knee and hip replacement

**Study country date**	**Inclusion patients (intervention: control)**	**Common treatment**		**Latest post-surgical follow up Outcomes Losses to follow up (intervention/ control) Risk of bias summary**
		**Intervention treatment (infiltrate volume) Further treatment (if given)**	**Control**	
**TOTAL HIP REPLACEMENT STUDIES**				
Aguirre et al. 2012 [34] Switzerland Not specified	THR (minimally invasive) N = 76 (38:38) 58:58 years 53:50% female	Spinal anaesthesia, PCA morphine		48 hours and to 3 months Intra-venous morphine consumption, VAS pain at rest and with motion, electrocardiogram, skin inflammation or infection, satisfaction. 4 (2:2) lost to follow up, 3/4 caused by catheter dislocation Low risk of bias
		20 ml solution containing 60 mg ropivacaine injected into wound before closure. Further continuous infusion through catheter	20 ml placebo injection of saline. Continuous infusion of saline through catheter	
Andersen KV et al. 2007 [16] Denmark 2005–2006	THR, OA, elective N = 80 (40:40) 62:61 years 90:85% female	Spinal, post-operative oral oxycodon hydrochloride as required		96 hours VAS pain, length of stay, time to mobilisation, side effects and complications, motor block (Bromage scale) 5 (2:3) patients lost to follow up Unclear (blinding of outcome assessment)
		101.5 ml solution containing 200 mg ropivacaine, 30 mg ketoralac and 0.5 mg epinephrine infiltrated during surgery. Further infiltrate through catheter intra-articularly 8 hours after surgery.	Epidural infusion of ropivacaine and morphine	
Andersen LJ et al. 2007 [27] Denmark Date not specified	THR, OA, uncemented, >80 years N = 37 (19:18) 62:64 years 84:56% female	Spinal anaesthesia, self-administered oral oxycodone as rescue medication		6 weeks VAS pain at rest and on leg raise up to 8 hours, WOMAC pain to day 4, WOMAC pain, stiffness and function after 1,2,4,6 weeks, EQ5D at 6 weeks, patient controlled analgesic use to discharge, adverse events 3 patients out of 10 not fitting inclusion criteria were identified retrospectively. No losses to follow up Low risk of bias
		151.5 ml saline solution containing 300 mg ropivacaine, 30 mg ketorolac, and 0.5 mg adrenaline infiltrated during surgery Further infusion through catheter on day 1.	Saline placebo infiltration Saline placebo infused through catheter on day 1	
Bianconi et al. 2003 [10] Italy Date not specified	THR and TKR (78% THR), elective N = 37 (18:19) 66:64 years 79:83% female	Spinal anaesthesia. Loading dose of intravenous morphine at end of surgery		72 hours VAS pain at 2,4,8,12,24,48,72 hours, opioid consumption (rescue medication), adverse events, length of hospital stay, patient satisfaction No losses to follow up Low risk of bias
		40 ml saline containing 200 mg ropivacaine infiltrated at end of surgery. Further ropivacaine infusion through catheter for 55 hours after closure. Intravenous saline infusion for 24 h after surgery.	No placebo infiltration during surgery. Saline infusion through catheter for 55 h after closure. Intravenous morphine plus ketorolac infusion for 24 h.	
Busch et al. 2010 [30] UK 2003–2005	THR, OA, age <80 years N = 64 (32:32) 61:65 years 50:54% female	General or spinal anaesthesia, PCA morphine		2 years VAS at rest and activity, morphine consumption (PCA), VAS satisfaction, complications, Harris Hip Score, WOMAC, length of hospital stay No losses to follow up Low risk of bias
		100 ml saline solution containing 400 mg ropivacaine, 30 mg ketorolac, 5 mg epimorphine, and 0.6 ml epinephrine (1:1000) infiltrated during surgery.	No placebo infiltration	
Dobie et al. 2012 [35] UK 2006–2007	THR, OA or RA N = 96 (50:46) 67:67 years 38: 52% female	Spinal, general, intravenous morphine after surgery as required.		6 days VAS at 24 h, morphine consumption, walking and stair test, mobilisation velocity and day, sit to stand test, home readiness, hospital stay, Iowa Level of Assistance Scale 4 (4:0) patients did not receive intervention as planned. Intention to treat results. Some data missing for 1 control Low risk of bias
		160 ml saline solution containing 200 mg levobupivacaine and adrenaline	No local infiltration	
Lee et al. 2009 [29] South Korea 2006–2007 Note: additional pre-emptive analgesia and epidural	THR, 13% OA, 72% Osteonecrosis N = 60 (30:30) 51:55 years 37:43% female	General anaesthesia		5 days VAS pain, ambulation, doses of parenteral analgesia, time to straight leg raise, complications No losses to follow up described Unclear (blinding of outcome assessment)
		Pre-emptive analgesia with oral Oxycodone and Celecoxib. Epidural anaesthesia. 90 ml saline solution containing 5 mg morphine, 40 mg methylprednisolone and 6.8 mg ropivacaine infiltrated during surgery. Post-operative oral Oxycodone and paracetamol.	No pre-emptive analgesia No epidural No injection during surgery Post-operative intravenous PCA and oral and injected analgesics as required	
Liu et al. 2011 [32] China 2008–2009	THR, OA, ASA I–III, <80 years N = 82 (41:41) 74:74 years 75:77% female	Spinal anaesthesia, PCA morphine		15 days and 9 months (range 6–12 months) for infection Morphine use, VAS pain, surgical outcome, mobilisation (time to straight leg raise and 90 degree flexion) 2 (1:1) lost to follow up Low risk of bias
		60 ml saline solution containing 5 mg morphine, 30 mg bupivacaine, 1 ml betamethasone and 0.5 ml epinephrine infiltrated during surgery.	60 ml saline infiltrated during surgery.	
Lu et al. 2010 [31] China Not specified	THR, primary N = 40 (20:20) No information on age and sex of patients	No description of common anaesthesia except PCA		48 hours VAS pain, use of PCA pump, adverse drug reactions No losses to follow up apparent Unclear (limited reporting)
		COX-2 inhibitor before surgery. 100 ml solution containing 0.15% ropivacaine infiltrated at end of surgery. COX-2 inhibitor after surgery	No COX-2 inhibitor before surgery. 100 ml saline placebo infiltrated at end of surgery. No COX-2 inhibitor after surgery	
Lunn et al. 2011 [33] Denmark 2009–2010	THR, >18 years N = 120 (60:60) 67:67 years 55:65% female	Spinal with or without general. Multimodal oral analgesia		8 hours and to discharge VAS pain at rest and during walking and passive hip flexion, Oxycodone consumption, complications No losses to follow up except “pain during walking” with 18 (11:7) lost to follow up Low risk of bias (except pain during activity: possible risk of bias due to large number of patients unable to complete test|)
		150 ml saline solution containing 0.2% ropivacaine and 10 μg/ ml epinephrine infiltrated during surgery.	150 ml saline placebo infiltrated during surgery.	
Murphy et al. 2012 [36] Ireland 2009–2010	THR, OA N = 91 (45:46) 57:54 years 49:38% female	Spinal, PCA opioid analgesia		72 hours WOMAC Pain, McGill Pain Questionnaire, VAS pain, morphine consumption, complications 13 (6:7) lost to follow up but some analyses used multi-level modelling to handle missing data Low risk of bias
		60 ml saline containing 150 mg levobupivacaine infiltrated during surgery.	60 ml saline placebo	
Parvataneni et al. Hip 2007 [28] USA 2005–2006	THR, OA N = 71 (35:36) 64:61 years 40:39% female	Spinal anaesthesia with or without FNB		3 months VAS pain, total narcotic dose, functional recovery including time to straight leg raise, side effects of narcotic use, patient satisfaction No losses to follow up reported Low risk of bias
		Intra-operative infiltration of 200–400 mg bupivacaine, 4–10 mg morphine sulphate 300 μg epinephrine, 40 mg methylprednisolone acetate, 75 mg cefuroxime and 22 ml saline. Total volume approximately 33 ml.	No infiltration during surgery Post surgical PCA	
Rikalainen-Salmi et al. 2012 [37] Finland 2009–2010	THR, OA, ASA I–III N = 60 (30:30) 65:66 years (followed up) 66:61% female (followed up)	Spinal, propofol if required, oxycodone rescue medication.		8 weeks NRS pain at rest and motion, oxycodone consumption, mobilisation, fulfilment of discharge criteria, satisfaction, adverse events and complications 3 (1:2) lost to early follow up. 7 (4:3) lost to long term follow up Low risk of bias
		101 ml solution containing 125 mg levobupivacaine, 30 mg ketorolac infiltrated during surgery 21 ml solution containing 100 mg levobupivacaine and 30 mg ketoralac administered through catheter on morning of first post-operative day	Intrathecal morphine No placebo infiltration Sham catheter attached to skin with 21 ml air administered on morning of first post-operative day (not inserted into joint )	
**TOTAL KNEE REPLACEMENT STUDIES**				
Affas et al. 2011 [50] Sweden 2007–2008	TKR, 77.5% OA, 22.5% RA, >18 years, ASA I–III, primary. N = 40 (20:20) 67:69 years 45:60% female	Spinal anaesthesia, PCA morphine		24 hours NRS pain intensity at rest and on movement, 24 hour morphine PCA consumption. No losses to follow up. Missing data analysis reported. Unclear risk of bias (blinding of outcome assessment)
		110 ml containing approximately 200 mg ropivacaine, 20 mg ketorolac and 0.33 mg epinephrine infiltrated during surgery. Further intra-articular infiltration through catheter after surgery.	Femoral nerve block. Intravenous ketorolac after surgery. No placebo infiltration.	
Andersen KV et al. 2010 [44] Denmark 2007–2008	TKR, >18 years N = 49 (24:25) 67:69 years 43:26% female	Spinal anaesthesia, PCA morphine		72 hours and to discharge. Infection to 30 days VAS/ NRS pain, morphine requirement, side effects and complications, time to achieve discharge criteria, length of stay, 9 (3:6) patients lost to follow up Unclear risk of bias (blinding of outcome assessment)
		151.5 ml saline solution containing 300 mg ropivacaine, 30 mg ketorolac and 0.5 mg epinephrine infiltrated during surgery. Further continuous infusion through catheter after closure.	Epidural infusion of ropivacaine. Post-operative intravenous ketorolac	
Busch et al. 2006 [38] Canada Date not specified	TKR, age <80 years N = 64 (32:32) 66:70 years 50:59% female	General or spinal anaesthesia, PCA morphine		6 weeks VAS at rest and activity, morphine consumption (PCA), VAS satisfaction, complications, Knee Society Score, WOMAC, length of hospital stay No losses to follow up Low risk of bias
		100 ml saline solution containing 400 mg ropivacaine, 30 mg ketorolac, 5 mg epimorphine, and 0.6 ml epinephrine (1:1000) infiltrated during surgery.	No placebo infiltration	
Carli et al. 2010 [45] Canada 2007–2008	TKR, OA, tricompartmental, cemented. N = 40 (20:20) 71:71 years 75:70% female	Spinal anaesthesia, PCA morphine		6 weeks Morphine consumption, NRS pain at rest and walking, functional capacity, ability to walk 30 m, physical activity, SF-12, WOMAC No losses to follow up Low risk of bias
		Solution of ropivacaine (0.2%), 1 ml of ketorolac (30 mg/ml), and 0.5 ml of epinephrine (1 mg/ml) with a total volume of 100 ml infiltrated during surgery. Further infusion through catheter after closure	Continuous femoral nerve block Saline injection Post-surgical infusion of saline	
Chen et al. 2012 [52] China 2008	TKR, OA, age <76 years. N = 81 (40:41) 66:65 years 75:78% female	Spinal anaesthesia, PCA morphine		15 days and infection to 6 months Total morphine consumption, VAS pain at rest and motion, time to straight leg raise and 90 degree flexion, adverse events including delayed infection 1 (0:1) patient lost to follow up Low risk of bias
		Intra-operative injection of a solution of magnesium sulphate (50 mg/kg) and 190 mg ropivacaine in normal saline to a volume of 100 ml.	Intra-operative intra-articular injection of 100 ml normal saline	
Essving et al. 2010 [46] Sweden 2007–2008	TKR, OA, ASA I–III, 20–85 years N = 48 (24:24) 72:70 years 54:54% female	General anaesthesia, PCA morphine		3 months PCA morphine consumption, VAS pain at rest and on knee flexion, time to home readiness, length of hospital stay, surgical outcome, functional outcome tests, Oxford Knee Score, EQ-5D, patient satisfaction, adverse events 1 (0:1) patient lost to follow up Low risk of bias
		116 ml saline containing 300 mg ropivacaine, 30 mg ketorolac and 0.5 mg epinephrine infiltrated during surgery. 50 ml saline containing 100 mg ropivacaine infiltrated before closure. Further injection of mixture 21 h after closure.	No placebo injections during surgery. Post-surgical injection of saline at 21 hours.	
Essving et al. 2011 [51] Sweden 2009–2010	TKR, OA, ASA I–III, age 40–85 years N = 50 (25:25) 71:71 years 64:60% female	Spinal anaesthesia, PCA morphine		3 months VAS pain, PCA morphine, verbal rating scale of satisfaction, functional tests, time to home readiness, Oxford Knee Score, EQ-5D, adverse events 2 (0:2) patients lost to follow up Low risk of bias
		Spinal plus intrathecal saline. Injection during surgery of 400 mg ropivacaine (160 ml), 30 mg ketoralac (1 ml) and 0.5 mg epinephrine (5 ml) Further infiltrate through catheter on day 1 and 2	Spinal plus intrathecal morphine No injection during surgery Post-surgical infusion of saline through catheter	
Fu et al. 2009 [42] China 2006–2007	TKR, OA, age <80y N = 80 (40:40) 69:68 years 75:78% female	Spinal anaesthesia, PCA morphine		15 days except ROM 90 days, infection 12 months Morphine consumption, VAS pain at rest and activity, ROM, time to straight leg raise, surgical outcomes, complications. No losses to follow up. Missing data imputation described Low risk of bias
		60 ml saline containing 5 mg morphine, 30 mg bupivacaine and 1 ml betamethasone infiltrated during surgery.	60 ml saline infiltrated during surgery	
Fu et al. 2010 [47] China 2008–2009	TKR, OA, age < 80 years N = 100 (50:50) 68:67 years 76:80% female	Spinal anaesthesia, PCA morphine		15 days except ROM at 90 days and infection to mean 7.5 months (range 6–9 months) VAS pain, morphine consumption (PCA and intramuscular) , time to straight leg raise and 90 degree flexion, surgical outcomes, adverse reactions No losses to follow up Low risk of bias
		Oral COX-2 inhibitor and tramadol 1 day before to 1 month after surgery 50 ml saline containing 5 mg morphine, 150 mg ropivacaine, 0.5 ml adrenaline and 1 ml betamethasone infiltrated during surgery.	Oral placebo 1 day before to 1 month after surgery 50 ml saline placebo infiltrated during surgery	
Han et al. 2007 1 and 2 [40] Korea 2005–2006 Note: 2 intervention groups	TKR, primary N = (30:30:30) 69:68:67 years 90:80:90% female	Spinal and epidural anaesthesia, PCA morphine		48 hours Incidence of booster PCA for 24 hours, amount of intra-venous tramadol, VAS pain at rest and exercising, side effects, range of flexion. No losses to follow up reported Low risk of bias
		1) 50 ml saline solution containing 300 mg ropivacaine, epinephrine (0.25 ml 1:200,000) and 5 mg morphine injected before wound closure. 2) 50 ml saline solution containing 300 mg ropivacaine and epinephrine (0.25 ml 1:200,000) injected before wound closure.	50 ml saline placebo	
Koh et al. 2012 [53] Korea 2008–2009	TKR, OA, unilateral N = 101 (49:52) 70:70 years 89:91% female	FNB, spinal anaesthesia, PCA morphine		7 days VAS pain at rest (day 1) and on movement (days 4 and 7), PCA opioid consumption, use of rescue medication, pain compared with expectations, functional recovery (straight leg raise and flexion), satisfaction, side-effects and complications, length of stay. 14 (4:10) did not receive treatment as planned. Results reported by intention to treat Low risk of bias
		50 ml saline containing ropivacaine 300 mg, morphine sulphate 10 mg, ketoralac 30 mg, 0.3 mg epinephrine, cefuroxime 750 mg injected/ infiltrated during surgery.	No placebo infiltration reported	
Krenzel et al. 2009 [43] USA 2007–2008	TKR, 96% OA elective. N = 67 (35:32), 1 patient with staged bilateral TKR included twice. 67:65 years 57:72% female	FNB, spinal anaesthesia, PCA fentanyl		24 hours PCA fentanyl consumption, NRS pain, functional tests, time to straight leg raise, ambulation distance, surgical outcomes, adverse events No losses to follow up Low risk of bias
		20 ml infiltration of 100 mg ropivacaine during surgery.	20 ml saline placebo infiltrated during surgery	
Mahadevan et al. 2012 [54] UK Not specified	TKR, OA or RA, unilateral N = 52 (26:26) 68:67 years 54:58% female	FNB, general anaesthesia, PCA morphine.		48 hours and to discharge VAS pain, morphine consumption, active ROM, length of hospital stay. No losses to follow up reported Low risk of bias
		25 ml saline containing 0.375% levobupivacaine infiltrated during surgery.	Sciatic nerve block No placebo infiltration reported	
Meftah et al. 2012 [55] USA 2010–2011	TKR, unilateral N = 90 (45:45) 65:67 years 64:64% female	Pre-emptive analgesia		3 days and to discharge. 6 months for infection, fracture and re-operation. Pain at rest and ambulation, readiness for discharge.1 (1:0) lost to all follow up, 6 (4:2) lost to readiness for discharge follow up Unclear (blinding of outcome assessment)
		45.1 ml saline solution containing marcaine (400–800 mg, morphine sulphate 8 mg, adrenaline 0.3 mg, antibiotic 750 mg, corticosteroids 40 mg injected during surgery.	FNB. PCA epiduralNo placebo injection reported	
Ng et al. 2012 [56] China 2008–2010 Note: crossover design. Patients having both knees replaced	TKR, OA N = 32 (16:16) surgeries but 16 patients only having 2 TKRs 3 months apart. 70:70 years 88:88% female	General anaesthesia, remifentanil infusion, PCA morphine		3 days and to discharge Pain score at rest and motion, total morphine consumption, Knee Society Score, ROM, quadriceps power, satisfaction, adverse events and complications.No losses to follow up reported Low risk of bias
		101.5 ml saline solution containing 300 mg ropivacaine, adrenaline 1 mg and triamcinolone acetonide 40 mg infiltrated during surgery. Femoral catheter inserted and saline infused.	Femoral nerve block. Wound infiltration with 101.5 ml saline.	
Parvataneni et al. Knee 2007 [28] USA 2005–2006	TKR, OA N = 60 (31:29) 69:71 years 45:52% female	Spinal anaesthesia with or without FNB		3 months VAS pain, total narcotic dose, functional recovery including time to straight leg raise, side effects of narcotic use, patient satisfaction No losses to follow up reported Low risk of bias
		Intra-operative infiltration of 200–400 mg bupivacaine, 4–10 mg morphine sulphate 300 μg epinephrine, 40 mg methylprednisolone acetate, 75 mg cefuroxime and 22 ml saline. Total volume approximately 33 ml.	No infiltration during surgery Femoral nerve block at end of surgery Post surgical PCA Effort to conceal allocation but no sham epidural	
Spreng et al. no iv injection 2010[48] Norway 2007–2009	TKR, unilateral, non-cemented, no patella resurfacing, age >17 years, ASA I–III. N = 68 (34:34) 67:66 years 61:67% female	Spinal. Propofol if indicated. PCA morphine		72 hours and to discharge VAS at rest and during knee flexion, morphine consumption, functional recovery, length of stay, satisfaction, mobilisation including walking distance, adverse events2 (1:1) lost to follow up Low risk of bias
		150 ml saline solution containing 150 mg ropivacaine, 0.5 mg epinephrine, 30 mg ketorolac and 5 mg morphine infiltrated during surgery. Knee injected through catheter with ropivacaine and ketorolac solution after 22–24 hours Intravenous injection with saline at 22–24 hours	48 hours of epidural analgesia as soon as spinal started to wear off No wound infiltration during surgery. No injections through sham catheter. No sham epidurals	
Spreng et al. with iv injection 2010 [48] Norway 2007–2009	TKR, unilateral, non-cemented, no patella resurfacing, age >17 years, ASA I–III. N = 68 (34:34) 67:66 years 61:67% female	Spinal anaesthesia, propofol if indicated, PCA morphine		72 hours and to discharge VAS at rest and during knee flexion, morphine consumption, functional recovery, length of stay, satisfaction, mobilisation including walking distance, adverse events 2 (1:1) lost to follow up Low risk of bias
		150 ml saline solution containing 150 mg ropivacaine and 0.5 mg epinephrine infiltrated during surgery. Also intravenous injection of 1 ml ketorolac (30 mg/ml) and 5 ml morphine (1 mg/ml).Knee injected with saline at 22–24 h (catheter) Intravenous injection with ketoralac at 22–24 h	48 hours of epidural analgesia as soon as spinal anaesthetic started to wear off. No wound infiltration during surgery. No injections through sham catheter	
Thorsell et al. 2010 [49] Sweden Not specified	TKR, OA or RA N = 85 (46:39) 69:72 years (followed up) 81:73% female (followed up)	Not specified, probable PCA		4 days and to discharge VAS pain, morphine consumption, satisfaction, mobilisation getting out of bed without assistance, walking with crutches), functional recovery, length of hospital stay 21 (13:8) patients lost to follow up data reported Possible bias (large uneven losses to follow up, group allocation by date of birth)
		Spinal anaesthesia 156 ml solution with 300 mg ropivacaine, 0.5 mg adrenaline and 30 mg ketorolac infiltrated during surgery. Further infiltrate through catheter intra-articularly on post-operative day 1.	Spinal or epidural analgesia No placebo infiltration reported Post-operative pain relief with ropivacaine infusion through epidural catheter.	
Toftdahl et al. 2007 [18] Denmark 2005–2006	TKR, OA with planned spinal anaesthesia 77 (40:37) 70:72 years 63:60% female	Spinal and after surgery immediate release oxycodone and intravenous morphine if required		4 days and to discharge NRS pain, opioid consumption, mobilisation (able to walk >3 metres, able to hold quadriceps tension for > 5 sec), length of hospital stay, adverse events and complications 4 (3:1) patients lost to follow up Unclear (blinding of outcome assessment)
		152 ml solution containing 300 mg ropivacaine, 30 mg ketorolac and 0.5 mg epinephrine infiltrated during surgery. Further infiltrate through catheter intra-articularly on day of surgery and post-operative day 1.	Femoral nerve block prior to spinal anaesthesia No placebo infiltration Post-surgical continuous femoral nerve block	
Vendittoli et al. 2006 [39] Canada 2003–2004	TKR, 95.2% OA N = 42 (22:20)Ages not specified 73:70% female	Spinal anaesthesia, PCA morphine		5 days and to discharge VAS pain at rest and during physiotherapy exercise, PCA morphine consumption, functional recovery, side effects No losses to follow up described Low risk of bias
		160 ml solution containing in total 400 mg ropivacaine, 30 mg ketorolac and 0.5 ml adrenaline (1:1000) infiltrated during surgery. Infiltrate through catheter intra-articularly on day1.	No placebo infiltration	
Zhang et al. 2007 [41] China 2006–2007	TKR, unilateral N = 60 (30:30) Overall 68 years 83:80% female	PCA morphine		72 hours VAS pain at rest and activity, functional recovery No losses to follow up described Unclear (blinding of outcome assessment)
		60 ml solution containing 0.25% bupivacaine, epinephrine (1:200,000) and 10 mg morphine infiltrated during surgery	No placebo injection	

TKR: total knee replacement, THR: total hip replacement , OA: osteoarthritis, RA: rheumatoid arthritis, PCA: patient controlled analgesia, FNB: Femoral Nerve Block, VAS: visual analogue scale, NRS: numerical response scale, ROM: range of motion, WOMAC: Western Ontario and McMaster Universities Arthritis Index.

#### Pain

**Figure 3:**
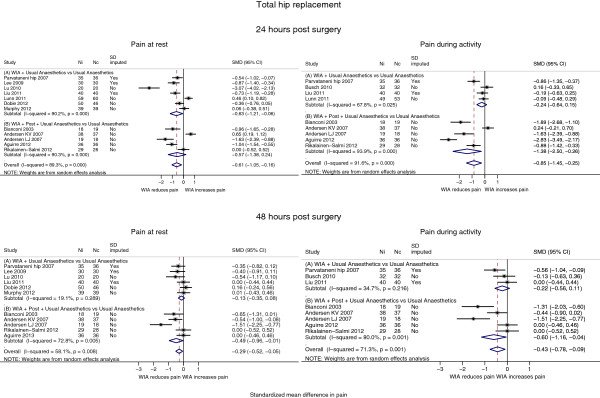
Total hip replacement: pain at rest and during activity by local anaesthetic infiltration grouping.

Results of meta-analyses including up to 12 studies [10,16,27-29,31-37], are summarised in Table 3 and Figure 3. In patients receiving local anaesthetic infiltration (A and B), there was strong evidence that pain was lower: at rest at 24 hours by SMD -0.61 (95% CI -1.05, -0.16; p = 0.008), and during activity by SMD -0.85 (95% CI -1.45, -0.25; p = 0.006). This reflected reduced pain at 24 hours at rest by an average of 12 points (95% CI 3, 21; p = 0.008), and during activity by 24 points (95% CI 7, 42; p = 0.006) on a 100 point scale. Average effect sizes at 48 hours were smaller for pain at rest, SMD -0.29 (95% CI -0.52, -0.05; p = 0.018) and during activity, SMD -0.43 (95% CI -0.78, -0.09; p = 0.014), corresponding to 5 and 10 points on a 100 point scale, respectively.

**Table 3:** Meta-analyses of pain and length of hospital stay by anaesthetic regimen compared with controls using a random effects model

**TOTAL HIP REPLACEMENT STUDIES**	**N**	**Measure**	**Pooled effect size**	**Confidence Interval**	**P-value**	**I**^**2 **^**(%)**	**τ**^**2**^
***(A + B) Any wound infiltration analgesia + usual anaesthesia vs Usual anaesthesia***							
Pain at rest at 24 h	12	SMD	-0.605	( -1.051, -0.160)	0.0078	89	0.541
Pain during activity at 24 h	9	SMD	-0.848	( -1.450, -0.246)	0.0058	92	0.765
Pain at rest at 48 h	11	SMD	-0.285	( -0.520, -0.050)	0.018	58	0.09
Pain during activity at 48 h	8	SMD	-0.432	( -0.776, -0.089)	0.014	71	0.171
Length of hospital stay	9	MD	-0.829	( -1.540, -0.118)	0.022	84	0.866
*(A) Wound infiltration analgesia + usual analgesia vs Usual anaesthesia*							
Pain at rest 24 h post-op	7	SMD	-0.633	( -1.208, -0.059)	0.031	90	0.529
Pain during activity 24 h post-op	4	SMD	-0.241	( -0.637, 0.155)	0.23	68	0.11
Pain at rest 48 h post-op	6	SMD	-0.134	( -0.348, 0.080)	0.22	19	0.014
Pain during activity 48 h post-op	3	SMD	-0.225	( -0.559, 0.109)	0.19	35	0.03
Length of Hospital Stay	5	MD	-0.257	( -0.622, 0.108)	0.17	14	0.029
*(B) Wound infiltration analgesia + post closure analgesia + usual anaesthesia vs Usual anaesthesia*							
Pain at rest 24 h post-op	5	SMD	-0.572	( -1.383, 0.240)	0.17	90	0.767
Pain during activity 24 h post-op	5	SMD	-1.378	( -2.499, -0.257)	0.016	94	1.525
Pain at rest 48 h post-op	5	SMD	-0.489	( -0.963, -0.015)	0.043	73	0.209
Pain during activity 48 h post-op	5	SMD	-0.599	( -1.158, -0.040)	0.036	80	0.319
Length of Hospital Stay	4	MD	-1.117	( -2.474, 0.239)	0.11	88	1.621
**TOTAL KNEE REPLACEMENT STUDIES**	**N**	**Measure**	**Pooled effect size**	**Confidence Interval**	**P-value**	**I2 (%)**	τ**2**
***(A + B) Any wound infiltration analgesia + usual anaesthesia vs Usual anaesthesia***							
Pain at rest at 24 h	12	SMD	-0.398	( -0.576, -0.219)	p < 0.001	32	0.032
Pain during activity at 24 h	12	SMD	-0.453	( -0.671, -0.235)	p < 0.001	54	0.078
Pain at rest at 48 h	12	SMD	-0.325	( -0.546, -0.103)	0.0041	56	0.084
Pain during activity at 48 h	11	SMD	-0.273	( -0.500, -0.046)	0.018	56	0.081
Length of hospital stay	8	MD	-0.866	( -1.622, -0.109)	0.025	77	0.805
*(A) Wound infiltration analgesia with no additional post closure analgesia + usual anaesthesia vs Usual anaesthesia*							
Pain at rest 24 h post-op	6	SMD	-0.248	( -0.452, -0.044)	0.017	14	0.009
Pain during activity 24 h post-op	6	SMD	-0.283	( -0.470, -0.096)	0.0031	0	0
Pain at rest 48 h post-op	6	SMD	-0.155	( -0.458, 0.148)	0.32	61	0.086
Pain during activity 48 h post-op	6	SMD	-0.077	( -0.263, 0.110)	0.42	0	0
Length of Hospital Stay	1	MD	0.092	( -0.890, 1.073)	0.85	100	p < 0.001
*(B) Wound infiltration analgesia + post wound closure analgesia + usual anaesthesia vs Usual anaesthesia*							
Pain at rest 24 h post-op	6	SMD	-0.587	( -0.829, -0.346)	p < 0.001	9	0.008
Pain during activity 24 h post-op	6	SMD	-0.693	( -1.152, -0.234)	0.0031	74	0.24
Pain at rest 48 h post-op	6	SMD	-0.52	( -0.778, -0.262)	p < 0.001	21	0.022
Pain during activity 48 h post-op	5	SMD	-0.594	( -0.997, -0.191)	0.0039	61	0.128
Length of Hospital Stay	7	MD	-1.023	( -1.822, -0.224)	0.012	76	0.761
*(C) Wound infiltration analgesia + post wound closure analgesia + usual anaesthesia vs Femoral nerve block + usual anaesthesia*							
Pain at rest 24 h post-op	3	SMD	0.253	( -0.514, 1.021)	0.52	81	0.37
Pain during activity 24 h post-op	3	SMD	0	( -0.317, 0.317)	1	0	0
Pain at rest 48 h post-op	2	SMD	0.254	( -0.429, 0.937)	0.47	67	0.166
Pain during activity 48 h post-op	2	SMD	-0.073	( -0.446, 0.299)	0.7	0	0
Length of Hospital Stay	2	MD	0.07	( -0.838, 0.978)	0.88	0	0
*(D) Wound infiltration analgesia + Femoral nerve block + usual analgesia vs Femoral nerve block + usual anaesthesia*							
Pain at rest 24 h post-op	3	SMD	-0.241	( -0.604, 0.122)	0.19	44	0.046
Pain during activity 24 h post-op	0						
Pain at rest 48 h post-op	1	SMD	-0.18	( -0.571, 0.211)	0.37	100	0
Pain during activity 48 h post-op	1	SMD	0.094	( -0.296, 0.485)	0.64	100	0
Length of Hospital Stay	1	MD	1.52	( 0.054, 2.986)	0.042	100	0
*(E) Wound infiltration analgesia + usual anaesthesia vs Femoral nerve block + usual anaesthesia*							
Pain at rest 24 h post-op	3	SMD	-0.076	( -0.632, 0.480)	0.79	69	0.166
Pain during activity 24 h post-op	3	SMD	0.159	( -0.869, 1.187)	0.76	90	0.741
Pain at rest 48 h post-op	2	SMD	0.056	( -0.300, 0.412)	0.76	0	0
Pain during activity 48 h post-op	2	SMD	-0.202	( -1.034, 0.631)	0.63	75	0.275
Length of Hospital Stay	2	MD	-0.069	( -0.634, 0.497)	0.81	0	0

N: Number of studies in meta-analysis. Two studies (Essving 2011, Parvataneni 2007 hip and knee) reported mean pain scores for the first 48 hours. These data were duplicated for the 24 and 48 hour outcomes. One study (Andersen LJ, 2007) reported WOMAC pain scores, a composite measure of pain taken at rest and during activity. These data were duplicated for rest and during activity outcomes.

In seven studies with no additional post-closure analgesia through a catheter or injection (A), patients receiving local anaesthetic infiltration reported lower pain at 24 hours at rest by SMD -0.63 (95% CI -1.21, -0.06; p = 0.031), equivalent to an average of 12 points lower pain. However, there was no strong evidence that the intervention had an effect during activity or at 48 hours.

In five studies where patients received further post-closure analgesia (B), pain was reduced on average at 24 hours during activity by SMD -1.38 (95% CI -2.5, -0.26; p = 0.016), equivalent to a 40 point decrease on a 100 point scale. Pain at 48 hours was reduced, on average, at rest by SMD -0.49 (95% CI, -0.96, -0.02; p = 0.043) and during activity by SMD -0.6 (95% CI -1.16, -0.04; p = 0.036) equivalent to 8 and 14 point decreases, respectively.

In one study, control patients received an epidural analgesia infusion [16]. Pain was lower for the duration of the epidural infusion, but at 48 hours pain was higher in the control group compared with the local anaesthetic infiltration group. In a study where control patients received additional intrathecal morphine, there was no difference in pain outcomes at any time point [37].

Heterogeneity measured by the I^2^ and τ ^2^ statistics was high, and separating the analysis for A and B groups did not appear to reduce this heterogeneity. Restricting the analysis to studies with low risk of bias gave a marginally smaller estimate of reduction in pain at 24 hours at rest by an average of SMD -0.49 (95% CI -0.89, -0.09; p = 0.017), but during activity average pain reduction appeared greater at SMD -0.99 (95% CI -1.64, -0.35; p = 0.003), corresponding to 28 points on a 100 point scale.

#### Opioid consumption

In all 11 studies reporting an outcome, opioid consumption was reduced in patients receiving local anaesthetic infiltration compared with controls [10,16,27,30-37]. This difference ranged from 12 to 92%. There was no suggestion of different effects in groups with or without additional analgesia through a catheter or injection.

In the studies where control patients received epidural or intrathecal analgesia, patients receiving local anaesthetic infiltration consumed 20% and 12% less morphine, respectively.

#### Mobilisation

Several different measures of mobilisation were reported. In three studies patients receiving local anaesthetic infiltration with no additional post-operative component achieved a straight leg raise earlier than control patients [28,29,32]. More patients were able to walk during the first post-operative day in two studies where additional post-operative analgesia was provided through a catheter [16,37]. In one study with no additional analgesia, with the exception of those with adverse events, all patients were mobilised on the first post-operative day [35]. However, in patients receiving local anaesthetic infiltration, walking speed over six metres at a two-day functional assessment was improved.

In one study, 35% of patients receiving local anaesthetic infiltration were able to walk after 8 hours compared with 87% of control patients receiving an epidural infusion. In the study where control patients received intrathecal morphine, 33% of these patients could walk further than 5 metres on the first post-operative day compared with 71% of patients receiving local anaesthetic infiltration.

#### Length of hospital stay

**Figure 4:**
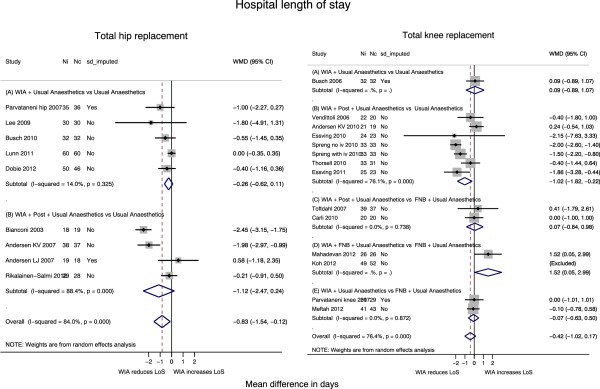
Length of hospital stay by local anaesthetic infiltration grouping.

As shown in Table 3 and Figure 4, patients receiving local anaesthetic infiltration spent an average 0.83 fewer days (95% CI 0.12, 1.54 days; p = 0.022) in hospital compared with controls. Benefit was largely driven by interventions with additional analgesia through a catheter or injection (B comparisons). Heterogeneity across studies was high (I^2^ = 84%), mainly in studies with additional post-operative analgesia.

When the comparison group received an epidural infusion [16], patients with local anaesthetic infiltration had on average a two day shorter hospital stay. In the study where the comparison group received intrathecal morphine [37], there was no clear difference in discharge times.

#### Complications

The Peto OR for a major complication in patients with local anaesthetic infiltration compared with controls was 0.30 (95% CI 0.05, 1.77; p = 0.18), but this is weak evidence, based on five major complications in 896 patients. Five deep infections were reported, four in local anaesthetic infiltration patients and one in controls, Peto OR 3.47 (95% CI 0.58, 20.81; p = 0.17). Four infections occurred in the 218 patients who received post-closure delivery of infiltrate through a catheter.

The incidence of vomiting was reduced in patients receiving local anaesthetic infiltration in five studies with 309 patients with data, Peto OR 0.46 (95% CI 0.27, 0.80; p = 0.006).

#### Long-term outcomes

Five studies reported long-term outcomes. Andersen and colleagues reported a trend for improved Western Ontario and McMaster Universities Osteoarthritis Index (WOMAC) pain scores at six weeks in local anaesthetic infiltration patients compared with controls [27]. At eight week follow up, Rikalainen-Salmi and colleagues reported no differences in mobilisation, intensity or duration of pain [37]. Parvataneni and colleagues reported that VAS pain scores were “comparable between groups” at 3 months [28]. Similarly, Aguirre and colleagues reported no difference in analgesic consumption or pain during normal daily activities between groups at 3 months [34]. Busch and colleagues reported a trend for improved WOMAC score at two years in local anaesthetic infiltration patients compared with controls [30].

### Total knee replacement

Overall there were 23 studies including 1,439 patients with TKR [18,28,38-56]. Study characteristics and our overall risk of bias assessment are summarised in Table 2. The mean number of patients randomised was 63 (range 32–101). We assessed that 17 studies were at low risk of bias and that five studies had unclear risk of bias based on uncertainty about blinding of outcome assessments. One study was assessed to be at high risk of bias due to a large uneven loss to follow up between randomised groups.

#### Pain

**Figure 5:**
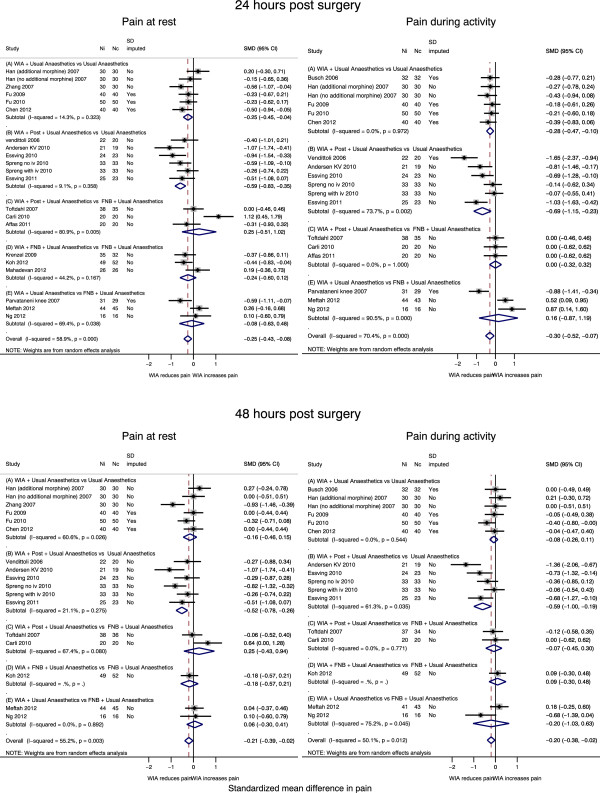
Total knee replacement: pain at rest and during activity by local anaesthetic infiltration grouping.

As shown in Table 3 and Figure 5, there was strong evidence that on average across 12 studies [39-42,44,46-48,51,52], patients receiving local anaesthetic infiltration (A + B studies) reported lower pain at rest compared with controls at 24 and 48 hours. For example, pain at rest at 24 hours and during activity at 48 hours was reduced by SMD -0.40 (95% CI -0.58, -0.22; p < 0.001) and SMD -0.27 (95% CI -0.50, -0.05; p = 0.018), respectively. This reflected reductions in pain at rest at 24 hours by an average of 10 points (95% CI 6, 15; p < 0.001) and during activity at 48 hours by 8 points (95% CI 1.5, 15; p = 0.018) on a 100 point scale.

Heterogeneity was moderate to low. When we restricted analyses to studies assessed as low risk of bias, pain outcome estimates were slightly attenuated towards zero.

We additionally performed separate analyses according to whether additional analgesia was delivered after wound closure through a catheter or injection. In the six studies with no further analgesia (A studies) [40-42,47,52], pain at 24 hours was lower at rest by SMD -0.25 (95% CI -0.45, -0.04; p = 0.017), and during activity by SMD -0.28 (95% CI -0.47, -0.10; p = 0.003). At 48 hours, pooled effect estimates favoured local anaesthetic infiltration but there was no strong evidence that the intervention was beneficial.

In six studies with additional analgesia delivered after wound closure (B studies) [39,44,46,48,51], pain was reduced on average at 24 hours at rest by SMD -0.59 (95% CI -0.83, -0.35; p < 0.001) and during activity by SMD -0.69 (95% CI -1.15, -0.23; p = 0.003). At 48 hours, pain was reduced at rest by SMD -0.52 (95% CI -0.78, -0.26; p < 0.001) and during activity by SMD -0.59 (95% CI -1.00, -0.19; p = 0.004).

In six studies comparing local anaesthetic infiltration with or without additional post-closure analgesia against femoral nerve block, there was no evidence for improvement in pain at any time point [18,28,45,50,55,56]. In three studies where both randomised groups received a femoral nerve block (D studies) [43,53,54], there was no evidence for added benefit of local anaesthetic infiltration for pain outcomes.

In eight comparisons between local anaesthetic infiltration with controls [38,39,44,46,48,49,51], additional ketoralac was included in the wound infiltrate. In seven comparisons with data [38,39,44,46,48,51], there was strong evidence that patients receiving additional analgesia in the infiltrate on average had lower pain compared with controls. For example, pain was reduced on average at rest at 24 hours by SMD -0.68 (95% CI -0.94, -0.42; p < 0.001) and during activity at 48 hours by SMD -0.59 (95% CI -1.01, -0.17; p = 0.006), equivalent to a reduction of 17 and 30 points respectively on a 100 point scale.

In four studies, control patients received either an epidural infusion [44,48,49] or intrathecal morphine [51]. Results of all studies supported a reduction in pain for patients receiving local anaesthetic infiltration compared with epidural or intrathecal morphine.

#### Opioid consumption

In all four studies reporting opioid consumption, this was reduced by 35–40% in patients receiving wound infiltration with no additional post-closure analgesia [38,42,47,52], and by 32–52% in three studies with additional post-closure analgesia, compared with controls [39,46,51].

In six studies where the control group or both groups received femoral nerve block, there was little difference in opioid consumption between randomised groups [18,28,43,45,50,53].

In four studies where patients receiving wound infiltration with further post-closure analgesia were compared with patients receiving epidural anaesthesia, there was no consistent difference between groups [44,48,49].

#### Mobilisation

Nineteen studies reported a mobilisation outcome. In four studies, patients receiving local anaesthetic infiltration had reduced time to achieve a straight leg raise by an estimated 44–50% [42,47,52] or were more likely to achieve a straight leg raise on the first post-operative day compared with control patients [28]. In two studies with femoral nerve block given to all patients, more patients receiving local anaesthetic infiltration were able to achieve a straight leg raise during the first post-operative day [43,53].

In four out of five studies, patients receiving local anaesthetic infiltration achieved better knee flexion [39,40,47,54]. In four studies [44,46,51,55], ambulation was part of discharge readiness criteria. These criteria were met earlier in local anaesthetic infiltration patients in three studies [44,46,51], but were similar in one study where control patients received a femoral nerve block [55].

Improvements to diverse walking goals were reported in patients receiving local anaesthetic infiltration in three studies where some or all of the comparison group patients received epidural analgesia [48,49]. When the comparison group or all patients received femoral nerve block, walking goals were achieved earlier after local anaesthetic infiltration in one study [18], with trends for benefit in two studies [43,45].

#### Length of hospital stay

Data on length of hospital stay were available for 8 studies comparing local anaesthetic infiltration with controls [38,39,44,46,48,49,51], of which seven had a post-closure analgesia component. As shown in Table 3 and Figure 4, length of hospital stay was reduced in patients receiving local anaesthetic infiltration and additional post-closure delivery (B studies) by 1.0 day on average (95% CI 0.2, 1.8 days; p = 0.012) compared with controls. In the one (A) study with no post-closure analgesia component there was no difference in length of hospital stay.

In three studies where the comparison group received femoral nerve block [18,45,55], there was no suggestion of a difference in length of stay. In one study in which all randomised patients received a femoral nerve block, the length of hospital stay was about 1.5 days shorter in the control patients who also received a sciatic nerve block [54].

In four studies where the control group received epidural analgesia [44,48,49], length of hospital stay was reduced in patients receiving local anaesthetic infiltration with the exception of one study in which the authors reported shorter time to fulfilment of discharge criteria [44].

#### Complications

Based on 11 events, the Peto OR for a major complication was 1.17 (95% CI 0.35, 3.86; p = 0.80) in patients receiving local anaesthetic infiltration compared with controls. There were two deep infections in intervention patients [18,44], and one in control groups [48], Peto OR 1.85 (95% CI 0.19, 17.83; p = 0.59). Two infections occurred in the 287 patients who received post-closure delivery of infiltrate through a catheter.

Excluding one intervention with additional morphine [40], there was evidence that the incidence of vomiting was lower in local anaesthetic infiltration patients compared with controls in eight studies with 548 patients [40,42,46-48,51,52], Peto OR 0.56 (95% CI 0.39, 0.80; p = 0.002).

#### Long-term outcomes

Five studies reported outcomes measured at six weeks [38,45], or three months [28,46,51]. Busch and colleagues showed a trend for improved pain at 6 weeks favouring the intervention group [38]. Parvataneni and colleagues reported comparable pain scores between groups at 3 months [28]. In the studies of Essving and colleagues, there were no differences between median Oxford Knee Scores at 3 months [46,51].

Carli and colleagues reported poorer WOMAC scores after 6 weeks in patients receiving local anaesthetic infiltration compared with the control group who received femoral nerve block [45].

## Discussion

Our systematic review and meta-analyses represent a comprehensive overview of evaluations of the effectiveness of peri-operative local anaesthetic infiltration in THR and TKR. Systematic reviews allow for a more objective appraisal than traditional narrative reviews [57], which are often biased in their selection of studies and thus may be unreliable in their recommendations of interventions [58]. Extensive efforts to acquire information from authors on unpublished outcomes and variance data allowed us to apply methods for meta-analyses of continuous and skewed outcomes and to produce more robust results for some outcomes than could be achieved with a purely narrative synthesis.

In conducting this systematic review we recognised the problems that can arise when small studies are included in meta-analyses [59]. In this review it is noteworthy that 28 out of 35 studies (80%) reported a power calculation. Review of studies with data largely collected in highly controlled conditions in the peri-operative and early post-operative period benefitted from low losses to follow up and more complete data. With the exception of one study where the authors acknowledged uneven losses to follow up due to inadequate protocols, the main risk of bias arose from uncertainty about blind outcome assessment. As most studies reported VAS pain and other self-reported outcomes, we believe that the evidence base on short-term outcomes is of reasonably good quality.

Pain after THR was reduced for patients receiving local anaesthetic infiltration, with patients experiencing less pain at rest at 24 hours and during activity at 48 hours equivalent to about 12 and 10 points on a 100 point pain intensity scale. In musculoskeletal settings, VAS pain changes of 11 [60], and 14 [61], are considered clinically significant [62]. Patients receiving local anaesthetic infiltration had lower pain levels after their THR, used less opioid medication and had a reduced incidence of vomiting and nausea. This may explain the early mobilisation and earlier discharge of patients who received local anaesthetic infiltration, irrespective of alternative pain management strategies. Opioid medication is a key strategy in the management of post-surgical pain but its use can delay mobilisation and rehabilitation [63].

Pain after TKR was also reduced for patients receiving local anaesthetic infiltration compared with controls, with less pain at rest at 24 hours and after 48 hours during activity, equivalent to reductions of about 10 and 8 points on a 100 point pain intensity scale. Opioid consumption was reduced compared with untreated control patients and there was a general observation of early mobilisation, reduced vomiting and nausea, and early hospital discharge. Inclusion of the non-steroidal anti-inflammatory agent ketoralac in the infiltrate seemed to enhance post-operative pain relief.

When compared with alternative regimens, results were not so clear. Pain levels after TKR were broadly similar when femoral nerve block was included in the general analgesia regimen or as a comparator. Likewise, opioid consumption was similar. There was some suggestion of benefit for earlier mobilisation, but length of hospital stay was not reduced in patients receiving local anaesthetic infiltration. Femoral nerve block is a well established method of providing analgesia after TKR and is associated with reduced opioid requirement and thus fewer side effects such as nausea and vomiting. However, femoral nerve block is associated with decreased quadriceps function for a time and an increased risk of falls [64,65].

In studies in patients receiving TKR where control groups received epidural or intrathecal analgesia, benefit was observed for reduced pain in patients receiving local anaesthetic infiltration. Opioid consumption did not differ between groups but mobilisation and hospital discharge were achieved earlier in patients receiving local anaesthetic infiltration.

The improvement in pain control and shorter hospital stay was greatest for patients receiving additional analgesia through a catheter or by injection. However, we observed a small but potentially important increase in rates of serious infection, particularly in patients receiving further infiltrate through a catheter post-wound closure. Across THR and TKR studies, there were eight cases of deep infection requiring surgical debridement or revision. Six of these were in patients randomised to wound infiltration analgesia with additional analgesia through a post-surgical catheter. Indeed, all patients with deep infection had been randomised to receiving a catheter although researchers reported that catheters in control groups were not inserted into the joint capsule. The overall rate of infection in patients with THR or TKR randomised into wound infiltration analgesia studies was 0.34% and in patients receiving an active catheter the rate was 1.4%.

Few studies in patients with THR or TKR reported long-term follow up of patients and results were equivocal. Acute post-operative pain is an important risk factor for long-term pain [66,67], and deserves appropriate consideration in future studies of peri-operative pain control.

Our study has limitations. Although meta-analyses performed were enhanced by extensive contact with authors, imputation was required for some measures of variability. The skewed nature of hospital stay required transformation under assumptions of a lognormal distribution [26]. For opioid consumption and mobilization there was insufficient consistency in measures reported to conduct anything but a systematic narrative overview. We noted a range of analgesia regimens, with different studies making different comparisons, particularly for TKR. We considered it unnecessary to make indirect comparisons between regimens, since direct evidence was available for all the comparisons of interest.

A further limitation of meta-analyses in a highly active field of research such as wound infiltration analgesia is that they may become out of date quickly. Their value is emphasised, however, in a widely cited example when studies of streptokinase in acute coronary heart disease were conducted long after a critical mass of evidence had been obtained from meta-analysis showing benefit for patients [68]. Prior to submission, we updated searches in December 2013 and identified 12 new studies, three in patients with THR and nine in patients with TKR. Our results for local anaesthetic infiltration in patients receiving THR were supported with reduced pain compared with untreated control [69-71], or similar pain compared with epidural analgesia [71]. The results of our meta-analyses in patients receiving TKR were also supported with improved early pain control in patients receiving local anaesthetic infiltration [72]; further pain reduction with added ketoralac [73,74] but not steroid [75]; and uncertainty when compared with femoral nerve block [76-80].

Our results show that local anaesthetic infiltration is effective in reducing short-term pain after THR and TKR when compared with no anaesthetic infiltration. The effect of local anaesthetic infiltration is enhanced with the addition of post-closure analgesia, although this needs to be considered in light of the infection risks associated with catheters [81]. In TKR, there may be no added benefit to femoral nerve block. Further studies are in progress to assess long-term effectiveness of local anaesthetic infiltration [11].

## Conclusions

Our systematic review and meta-analysis shows that inclusion of local anaesthetic infiltration in a multi-modal anaesthesia regimen is effective in reducing short-term pain and hospital stay in patients receiving THR and TKR. Enhanced pain control was observed when additional analgesia was provided after wound closure through a catheter but benefit should be weighed against a possible infection risk. For patients with TKR, inclusion of the non-steroidal anti-inflammatory agent ketoralac in the infiltrate seemed to enhance pain relief. There was no evidence of pain control additional to that provided by femoral nerve block in patients receiving TKR. Few studies reported long-term outcomes and future research should assess whether local anaesthetic infiltration can affect the development of long-term post-surgical pain.

## Abbreviations

THR: Total hip replacement; TKR: Total knee replacement; RCT: Randomised controlled trial; SMD: Standardised mean difference; MD: Mean difference; VAS: Visual analogue scale; OR: Odds ratio; WOMAC: Western Ontario and McMaster Universities Osteoarthritis Index.

## Competing interests

The authors declare that they have no competing of interests.

## Authors’ contributions

ADB, EM, AWB and MP designed the study and produced the search strategy. EM and ADB performed the systematic review as second and first reviewers respectively, conducted the searches, screened abstracts and titles, assessed inclusion and exclusion criteria, produced data collection forms and extracted data, assessed study quality and contacted authors. AWB and MP advised on inclusion/ exclusion criteria and subgroup analyses. HEJ provided statistical guidance. EM and HEJ performed meta-analyses of continuous and skewed outcomes (pain and length of stay). KTE and ADB collected complications data and performed meta-analyses of complications. EM, ADB and HEJ drafted the article with critical revisions from AWB, MP and KTE. All authors read and approved the final manuscript.

## Pre-publication history

The pre-publication history for this paper can be accessed here:

http://www.biomedcentral.com/1471-2474/15/220/prepub

## Supplementary Material

Additional file 1 PRISMA checklist. Click here for file

Additional file 2 Cochrane risk of bias table (✓ low risk, X risk; ~ no reason to assume bias). Click here for file
