# Supplementary material for: Local anaesthetic infiltration for peri-operative pain control in total hip and knee replacement: systematic review and meta-analyses of short- and long-term effectiveness
Source: BMC Musculoskelet Disord. 2014 Jul 5;15:220. doi: 10.1186/1471-2474-15-220 (PMC4118275; doi:10.1186/1471-2474-15-220)
Supplement: Additional file 2 — Cochrane risk of bias table (✓ low risk, X risk; ~ no reason to assume bias). [file 1471-2474-15-220-S2.docx]

# Supplementary file2. Cochrane risk of bias table (✓ low risk, 🗶 risk; ~ no reason to assume bias)

|  | Random sequence generation | Allocation concealment | Blinding of participants/ personnel | Blinding of outcome assessment | Incomplete outcome addressed | Lack of selective reporting | Lack of other bias | Our evaluation |
| --- | --- | --- | --- | --- | --- | --- | --- | --- |
| **TOTAL HIP REPLACEMENT** | | | | | | | | |
| Andersen KV et al. 2007[[14]](#_ENREF_14) | ✓ | ✓ | 🗶 | 🗶 | ✓ | ✓ | ✓ | Unclear |
| Lee et al. 2009[[29]](#_ENREF_47) | ~ | ~ | 🗶 | 🗶 | ✓ | ✓ | ✓ | Unclear |
| Lu et al. 2010[[31]](#_ENREF_49) | ~ | ~ | ~ | ~ | ~ | ~ | ~ | Unclear |
| Aguirre et al. 2012[[25]](#_ENREF_43) | ~ | ~ | ✓ | ✓ | ✓ | ✓ | ✓ | Low |
| Andersen LJ et al. 2007[[26]](#_ENREF_44) | ✓ | ✓ | ✓ | ✓ | ✓ | ✓ | ~ | Low |
| Bianconi et al. 2003[[8]](#_ENREF_10) | ✓ | ✓ | 🗶 | ✓ | ✓ | ✓ | ✓ | Low |
| Busch et al. 2010[[27]](#_ENREF_45) | ✓ | ~ | 🗶 | ✓ | ✓ | ✓ | ✓ | Low |
| Dobie et al. 2012[[28]](#_ENREF_46) | ✓ | ✓ | 🗶 | ✓ | ✓ | ✓ | ✓ | Low |
| Liu et al. 2011[[30]](#_ENREF_48) | ✓ | ✓ | ✓ | ✓ | ✓ | ✓ | ✓ | Low |
| Lunn et al. 2011[[32]](#_ENREF_50) | ✓ | ✓ | ✓ | ✓ | ~ | ✓ | ✓ | Low (except pain during activity) |
| Murphy et al. 2012[[33]](#_ENREF_51) | ~ | ✓ | 🗶 | ✓ | ✓ | ✓ | ✓ | Low |
| Parvataneni et al. 2007 [[34]](#_ENREF_38) | ~ | ~ | 🗶 | ✓ | ✓ | ✓ | ✓ | Low |
| Rikalainen-Salmi et al. 2012[[35]](#_ENREF_52) | ✓ | ✓ | 🗶 | ✓ | ✓ | ✓ | ✓ | Low |
| **TOTAL KNEE REPLACEMENT** | | | | | | | | |
| Affas et al. 2011[[36]](#_ENREF_23) | ✓ | ✓ | 🗶 | 🗶 | ✓ | ✓ | ✓ | Unclear |
| Andersen KV et al. 2010[[37]](#_ENREF_24) | ✓ | ✓ | 🗶 | 🗶 | ✓ | ✓ | ~ | Unclear |
| Meftah et al. 2012[[49]](#_ENREF_36) | ~ | ~ | 🗶 | ~ | ✓ | ✓ | ✓ | Unclear |
| Toftdahl et al. 2007[[15]](#_ENREF_15) | ~ | ✓ | 🗶 | 🗶 | ✓ | ✓ | ✓ | Unclear |
| Zhang et al. 2007[[54]](#_ENREF_42) | ~ | ~ | 🗶 | ~ | ✓ | ✓ | ✓ | Unclear |
| Busch et al. 2006[[38]](#_ENREF_25) | ✓ | ~ | 🗶 | ✓ | ✓ | ✓ | ✓ | Low |
| Carli et al. 2010[[39]](#_ENREF_26) | ✓ | ✓ | ✓ | ✓ | ✓ | ✓ | ✓ | Low |
| Chen et al. 2012[[40]](#_ENREF_27) | ✓ | ✓ | ✓ | ✓ | ✓ | ✓ | ✓ | Low |
| Essving et al. 2010[[41]](#_ENREF_28) | ✓ | ~ | 🗶 | ✓ | ✓ | ✓ | ✓ | Low |
| Essving et al. 2011[[42]](#_ENREF_29) | ✓ | ✓ | 🗶 | ✓ | ✓ | ✓ | ~ | Low |
| Fu et al. 2009[[43]](#_ENREF_30) | ✓ | ✓ | ✓ | ✓ | ✓ | ✓ | ✓ | Low |
| Fu et al. 2010[[44]](#_ENREF_31) | ✓ | ✓ | ✓ | ✓ | ✓ | ✓ | ✓ | Low |
| Han et al. 2007 1[[45]](#_ENREF_32) | ✓ | ~ | ~ | ✓ | ✓ | ✓ | ✓ | Low |
| Han et al. 2007 2[[45]](#_ENREF_32) | ✓ | ~ | ~ | ✓ | ✓ | ✓ | ✓ | Low |
| Koh et al. 2012[[46]](#_ENREF_33) | ✓ | ✓ | 🗶 | ✓ | ✓ | ✓ | ✓ | Low |
| Krenzel et al. 2009[[47]](#_ENREF_34) | ~ | ~ | ✓ | ✓ | ✓ | ✓ | ✓ | Low |
| Mahadevan et al. 2012[[48]](#_ENREF_35) | ✓ | ✓ | 🗶 | ✓ | ✓ | ✓ | ✓ | Low |
| Ng et al. 2012[[50]](#_ENREF_37) | ✓ | ~ | ✓ | ✓ | ✓ | ✓ | ✓ | Low |
| Parvataneni et al. 2007 [[34]](#_ENREF_38) | ~ | ~ | 🗶 | ✓ | ✓ | ✓ | ✓ | Low |
| Spreng et al. no iv injection 2010[[51]](#_ENREF_39) | ✓ | ✓ | 🗶 | ✓ | ✓ | ✓ | ✓ | Low |
| Spreng et al. iv injection 2010[[51]](#_ENREF_39) | ✓ | ✓ | 🗶 | ✓ | ✓ | ✓ | ✓ | Low |
| Vendittoli et al. 2006[[53]](#_ENREF_41) | ✓ | ~ | 🗶 | ✓ | ✓ | ✓ | ✓ | Low |
| Thorsell et al. 2010[[52]](#_ENREF_40) | ~ | ~ | 🗶 | ~ | 🗶 | ✓ | ✓ | Possible bias (large uneven losses to follow up) |

Random sequence generation (selection bias); Allocation concealment (selection bias); Blinding of participants and personnel; Blinding of outcome assessment (detection bias); Incomplete outcome data addressed (attrition bias); Lack of selective reporting (reporting bias); Lack of other sources of bias
